# The Relationship between City Size and Carbon Monoxide (CO) Concentration and Their Effect on Heart Rate Variability (HRV)

**DOI:** 10.3390/ijerph18020788

**Published:** 2021-01-18

**Authors:** Diana Saadi, Emanuel Tirosh, Izhak Schnell

**Affiliations:** 1 Porter School of the Environmental and Earth Sciences, The Faculty of Exact Sciences, Tel Aviv University, Tel-Aviv-Jaffa 66978, Israel; 2 Bnei Zion Medical Center, (Emeritus) the Rappaport Family Faculty of Medicine, The Technion, Israel Institute of Technology, Haifa 23774, Israel; emi.tirosh@gmail.com; 3 Department of Geography and Human Environment, Tel Aviv University, Tel-Aviv-Jaffa 66978, Israel; schnell@post.tau.ac.il

**Keywords:** carbon monoxide (CO), autonomic nervous system (ANS), heart rate variability (HRV), air pollution, urban environments

## Abstract

Generally, larger cities are characterized by traffic congestion, which is associated with higher concentrations of pollution, including Carbon Monoxide (CO) pollution. However, this convention requires empirical support on the basis of accurate and reliable measurements. In addition, the assessment of the effect of CO on the autonomic nervous system (ANS), as measured by heart rate variability (HRV), has yielded conflicting results. A majority of the (few) studies on the topic have shown that increases in CO concentration of up to about 10 parts per million (ppm) are associated with a decrease in stress and risk to health in subjects. Beyond the hypothesis postulating city size as a determinant of increased CO concentration, the hypothesis proposing a causal link between CO concentration and HRV balance also requires empirical support. This article compares CO concentrations in a large metropolis with those in a small town, analyzing the relationship between CO and the HRV responses of young women in terms of city size. Four different types of environments were compared, taking into account mediating variables. The study participants spent 35 min in selected environments (a city center, a residential environment, a park, and a home) wearing Polar devices to measure HRV, and portable devices to measure noise thermal load and CO. The average concentrations of CO in each environment were calculated, along with the time distribution of the CO concentration, and the regression slopes between the concentrations of CO and the ANS balance, as measured by the low frequency power/high frequency power ratio (LF/HF) expressed as an HRV index. The results show that, regardless of size, the cities measured were all characterized by low levels of CO, far below the maximal accepted threshold standards, and that urban residents were exposed to these concentrations for less than half of the daytime hours. Furthermore, in contrast to the common view, larger cities do not necessarily accumulate higher concentrations of CO compared to small cities, regardless of the level of transport congestion. This study confirms the findings of the majority of the other studies on the subject, which showed a decrease in stress (as measured by HRV) as a result of an increase in CO concentrations below 7 ppm. Finally, following the assessment of the differential contribution attributed to the different environmental factors, it appears that noise, thermal load, and congestion all contribute more to a higher level of HRV balance than CO. This finding highlights the importance of a multivariable approach to the study, and a remediation of the effect of environmental factors on stress in urban environments.

## 1. Introduction

Carbon Monoxide (CO) is considered to be a principal pollutant in cities [1]. It is one of the five pollutants included in the Air Quality Index [2]. In most cities, vehicle traffic is the main source of air pollutants, including CO. However, with the improvements in the quality of fuel, CO levels are expected to reduce. Nevertheless, it is also predicted that CO concentrations will increase in proportion to the city size and the number of cars in the city. Furthermore, it is claimed that exposure to CO, which is a toxic agent, is associated with increased rate of headaches, dizziness, and nausea. It may also cause inflammation and oxidative stress, with a concomitant effect on the central autonomic nervous system (ANS), and in consistent and high concentrations it can cause increased mortality [3]. However, a review by Tirosh and Schnell [4] argued that increases in concentrations of CO, when controlling for other air pollutants, in fact moderate the increase in Heart Rate Variability (HRV) as an index of the balance of the autonomic nervous system. The results are inconsistent, however, across the few relevant studies conducted up until 2015 [4]. As recently as 2019, Tang et al. [5] observed that very few studies focused on the relationship between transportation, air pollution, and HRV, overlooking the effect of vehicle traffic on CO concentrations and the consequent effect on HRV [5]. In this context, the following three questions may arise: (1) To what extent has CO concentration remained low in contemporary cities? (2) To what extent do CO concentrations increase in accordance with city size and traffic congestion? (3) To what extent can an increase in CO concentrations in the lower ranges, which characterizes urban environments, be associated with increases in risks to health, as reflected by HRV? The objective of this study is to identify the exceptional behavior of CO with regard to health risks, both in terms of concentrations in cities and its effect on the people exposed to it.

### Background

Gaseous air pollution in cities mainly originates from the incomplete combustion of fuels by cars, factories, and heating. In China, more than 75% of the air pollution originates from these sources [6]. In Israel, more than 90% of the CO emissions come from vehicles, other than for a small number of cities characterized by high industrial activity concentrations. Consequently, in the two cities investigated in the present study—Tel Aviv (TA), a metropolis, and Afula, a small city—at least 90% of all air pollution, including CO, originates from vehicle traffic [7].

Several studies in the literature have considered the association between the traffic load and the concentration of air pollution in cities [5]. While these studies generally show an association between city size and morphology and levels of air pollution, the results are conflicting [8,9]. For example, Liu et al. [6] found city size to be significantly associated with concentrations of air pollution, over and above the morphological characteristics of the urban environment. However, this study did not focus on the association between city size and CO [6]. A study by Molnár et al. (2020) confirmed the independent effect of city size on air pollution, as measured by the Air Pollution Tolerance Index [10]. However, the study did not isolate the specific air pollutants contributing to this association. For example, the compactness of cities may, on the one hand, reduce the use of motor transportation, and thus air pollution; on the other hand, the same factor may increase concentrations of CO and other pollutants in morphological pockets [6,8,11]. The size of green spaces in a city may be associated with population dispersal and urban sprawl, thereby reducing both the compactness and concentrations of CO. Considered from another perspective, while green environments reduce air pollution, sprawl increases the use of private cars and travel distances, potentially contributing to increased CO concentrations [12]. Reviewing these potential contradictions, Liu et al. [6] concluded that the relevant characteristics of urban morphology must be explicitly defined in any analysis of the associations between city size and morphology and air pollution [6]. None of the studies cited above focused on the association between city size, the CO concentration in the city, and its effect on human health. Unlike these studies, our study is unique in focusing on the effect of CO on stress and the risk to health, highlighting the complex relationships between cities’ characteristics and the concentrations of CO in them.

In the index of air pollutants, CO differs from the other gasses because it is unstable and reacts with oxygen to produce CO_2_. Therefore, variations in CO concentration may not be related to city size, but rather to other factors such as congestion and city morphology. A study by Helfter, et al. [13] confirmed that low levels of CO are widely distributed in London, with somewhat higher concentrations in the winter [13].

The CO levels in Israel are currently monitored from stationary stations maintained by the Ministry for the Protection of the Environment. These stations are generally located on the roofs of high-rise buildings, some distance from major roads. As such, the monitored levels of CO concentration may not be accurate and may potentially be underestimated [14,15,16]. Recent studies have shown significant differences in the levels of air pollutants (including CO) between different sites, depending on the morphology of the location and the wind direction associated with the street layout and other salient factors [16,17]. Several studies have shown that the exposure of pedestrians to air pollution is proportional to the intensity of traffic along the adjacent streets, and that exposure to CO declines steeply in line with the distance from the sources [16].

HRV is considered to be a reliable index measure of stress and the risk to health [18,19], and it has been empirically validated in many studies [20]. HRV reflects the human ability to cope with environmental stressors that psychologically and physiologically challenge ANS activity. The exposure to stressors is typically associated with the decreased activity of the parasympathetic tone, and the increased activity of the sympathetic tone [21]. These changes in ANS activity reflect stress in the short term, and the elevated risk of a wide range of health problems in the long term [22].

Inaccurate CO measurements have cast doubt on the documented correlations between CO and HRV. A review by Tirosh and Schnell [4] identified 25 articles that analyzed the correlation between CO concentration and HRV. Most of these studies measured CO from stationary stations, located at a distance from the major roads and sidewalks where people perform their daily activities, and some distance from the subjects tested in these studies. Of these studies, only six measured CO from a location that was relatively close to the subjects being measured in the research. Out of the six studies, four reported negative correlation coefficients between CO and LF/HF in response to exposure to low levels of CO. In these studies, increases in CO concentrations, up to about seven parts per million (ppm), were associated with an improved ANS balance, as reflected by HRV. In these cases, the levels of LF/HF declined with the increase in the CO concentration. These results are consistent with the studies showing a decline in the levels of LF/HF with exposure to acute CO concentrations [23]. In a previous study, Tang et al. (2019) showed that, while an increase in the concentrations of dust contributes to increased levels of LF/HF, an increase in CO levels caused a decrease in the levels of LF/HF [24]. In a review of the association between low CO concentrations and HRV by Tirosh and Schnell [4], only two studies which employed different methodologies and different CO concentration ranges deviated from this trend. In the present study, we measured the subjects’ exposure to CO in the immediate vicinity of everyday life—in streets, neighborhoods, parks, city centers, and indoor environments like the family home [4]. We measured both the exposure to CO and the HRV of the subjects while they were in situ in these everyday environments.

It appears that the mean CO concentrations remain low in cities, with traffic being the main source of CO. CO concentrations have an effect on the autonomic nervous system, but in moderating levels of LF/HF, unlike the opposite effects evinced by other air pollutants. These conclusions are drawn from a handful of studies, indicating the need for empirical studies to further unpick this intricate relationship. The present study seeks to validate the relationship by making a comparison between the concentrations of CO and HRV in two cities, one large and one small, in Israel. Because the analysis in this article is based on two different studies in the two cities, some adjustments are required in order to validate this comparison.

We hypothesize that: (1) The mean concentration of CO in cities remains low, indeed significantly below the accepted standards. (2) Larger cities are characterized by higher CO concentrations. (3) Increases in the concentrations of low-level CO (up to 6 ppm) are associated with decrease in LF/HF.

## 2. Research Methods

The study is based on data derived from two separate empirical projects: one in Tel-Aviv, a metropolitan area in the center of Israel, and the other in Afula, a small city in northern Israel. TA has a population of approximately 460,000 people, and is part of a wider metropolitan area with a total population of 2.5 million. Afula has about 54,000 inhabitants. TA. is four times denser compared to Afula (8894 compared to 1860 per km). TA. is ranked at the eight tens of cities in Israel, while Afula is ranked at the fifth tens. Afula has less space for urban parks, but much more space for forests at its outskirts compared to TA. The park space in TA. comprises 4.6% of the urban space, and in Afula only 0.8%. However, in Afula, forest spaces form 15% of the municipal space, compared to almost zero in TA.

In both experiments, an ecological approach was used to test the effect of human exposure to a set of environmental factors on ANS, as measured by HRV, across four types of urban environments: home, residential neighborhood, city center, and park. In the present study, data relating to CO concentrations and HRV were extracted. Similar environments in the two cities were chosen. Although the measurements were derived from two different studies conducted two years apart, they are comparable in the sense that both measurements used similar methodologies, procedures, equipment, and climatic conditions. Despite the time gaps, the mean physiological equivalent temperature PET values were similar and within the range of comfortable weather (24 °C, 3.7 SD in TA; 22 °C, 1.5 SD in Afula). The concentrations of CO in the atmosphere—as measured by the stationary stations in Afula and Tel Aviv during the days of the experiments—remained at zero or close to zero, such that it supports the postulated general comparability of the two cities in terms of carbonic pollutants. The two cities have a similar level of compactness (9/10 in the compactness scale of Israeli cities), but with significant differences in traffic congestion, which is significant as this is deemed the sole source of air pollution in general, and CO in particular (Table 1).

*Participants*: We measured the effect of immediate exposure to CO, noise, thermal load, and HRV on 20 young female subjects in Tel Aviv, and 24 young female subjects in Afula. All were non-smoking, healthy women aged between 26 and 40 years old. None of the participants were on any form of medication, and none drank alcohol. Several studies have identified gender-related differences in HRV results, with women’s ANS being more sensitive to environmental factors [25,26]. The focus on women allowed more sensitivity in the expected HRV responses.

*Research flow*: The research study was conducted in three stages. First, two indices of personal exposure to CO were determined: the average concentration and the percentage of time exposed to any level of CO. Second, a comparison of the exposure to CO was calculated for the two cities. Third, regressions between CO and LF/HF were calculated for the six outdoor environments, controlling for noise and thermal load.

*Study sites*: The participants in Tel-Aviv carried the devices on their bodies for a period of 48 continuous hours, across a wide range of sites and along a pre-planned route, which was similar for all subjects. In Afula, participants carried the devices for half a day, in four selected environments: a park, a residential neighborhood, a city center site, and a home. The order of the sites was randomly selected [27]. In both cities, the participants spent 35 min at each station, sitting in the station with the devices attached to their bodies. Because we were measuring the immediate responses to environmental nuisances by HRV, with a response latency of 2–4 s, a short stay of half an hour was considered sufficient to record the immediate responses. During their stay at the site, the participants completed a short questionnaire concerning their feelings of comfort at the time. A washout period of 15 min was kept between each environment; the participants were transported between the sites in an inner-circulated air-conditioned car, with a temperature maintained at 22 °C.

In order to enable a comparison between the cities, the researchers only extracted the results derived from a 35-min stay in the four referenced environments recorded in TA. Of all the recorded CO measurements in TA, the researchers selected measurements from three city centers and three parks, in different locations at least half a kilometer apart, to represent the metropolitan area. Thus, the subjects were monitored in the real-life environments where they usually performed their daily lives.

*Procedure*: The experiments were conducted in stages, with six subjects taking part in each stage. They all followed the same route at the same time, but with no interactions between themselves. The measurements in TA and Afula were performed on different dates; however, the temperature differences between the cities were marginal. The measurements were all taken during the afternoon, a time of day with some traffic congestion but not as intensive as peak rush hour traffic. Each of the subjects carried a Polar device to measure HRV. They were accompanied by a researcher who was available to provide support in the event of any problems related to the monitoring gear, to register unusual events along the course of the experiment, and to make sure that the subjects adhered to the research protocols. In total, eight stages (four in each city) were conducted, half during the summer and half during the winter.

*Instruments*: A Polar 810i monitor was attached to each subject in order to continuously measure their heart beat rhythm, as a basis for the calculation of HRV indices. The device automatically extracted the main frequency and time domain indices of HRV. In measuring the frequency domain, low power frequencies (LF) were conceptualized as results between 0.04–0.15 Hz, and high power frequencies (HF) were conceptualized as results between 0.15–0.4 Hz. At the end of each stage, the researcher uploaded the data from the devices to a portable computer. The extraction of the data from the Polar was supported by a Protrainer 5 program. The data processing was performed by Kubios, which automatically cleaned the deviant data and calculated the main indices of the HRV.

The accompanying investigator carried an electrochemical Pac III sensor that was used to measure concentrations of CO, and a Quest pro DL dosimeter with a range of 40–110 d(B) and a resolution of 0.1 d(B). The average noise per minute was the basis for the calculation of the average noise per site. The thermal load was measured by Fourier micro log and Kestrel, which measures temperature relative to humidity and wind. The radiant temperature was recorded according to measurements from the closest stationary station maintained by the Ministry for the Protection of the Environment; the results were adjusted to reflect the degree of shade in the tested environment, as evaluated by the accompanying researcher during the tests. The Physiological Equivalent Temperature (PET) index was calculated by Ray Man [28], together with the other data about the subjects’ body characteristics taken from the subjects before the experiment.

The data were continuously collected for each campaign, and the average results for each station were calculated accordingly. The devices were calibrated according to the manufacturer’s instructions before the commencement of each set of readings. Further details are described in our previous study [29]. The same devices were used for all of the experiments.

The social load was determined from the participants’ responses, recorded on a 1 to 100 visual analog scale (VAS) with a color ladder ranging from light green (1) to deep red (100), indicating the sense of discomfort experienced as a result of the presence of others in the immediate vicinity. The responses were recorded every half hour at each of the sites where the subjects stayed, and once along the way between the stations. The validity of this protocol has been documented in an earlier study related to HRV and ethnicity conducted by our laboratory [29,30]. In these earlier studies, social discomfort was highly correlated with HRV indices.

*Analysis*: The analysis was conducted in two stages. First, for TA and Afula, the average concentrations of CO for the four types of environments (home, residential, city center, and park) were calculated, and the percentage of time that the subjects were exposed to any level of CO in each of the types of environment was calculated accordingly.

Second, the regressions between the CO concentrations and the subjects’ HRV levels were calculated for each environment in TA and Afula, controlling for the effects of noise and thermal load. Six types of curve estimations were run—linear, cubic, power, logistic, growth, and exponential—in order to characterize the curve that most accurately predicted the HRV responses to CO. The cubic function best predicted LF/HF, with the linear function as a second choice. We chose the linear function for the analysis as it is more amenable for calculations without giving up much predictive power. Finally, we employed a multiple regression in order to estimate the effects of CO and city size on LF/HF.

## 3. Results

Table 1 presents the population and traffic characteristics of the cities. The population of TA is eight times that of Afula, with ten times the total number of cars recorded at the cities’ main entrances. Considering the fact that TA has a significantly larger total area, the traffic congestion in TA can be calculated as 3.7 times greater than that of Afula.

The CO concentrations in the two cities remained extremely low, with the mean results not exceeding 5 ppm per half an hour (Table 2). As expected, the mean concentration of CO was highest in the two city centers, regardless of their size. The CO concentration in TA city center was lower than that in Afula. This may be partly due to the older age of the cars in Afula (Table 1). Lower concentrations of CO were recorded in the residential neighborhoods, with much lower concentrations for Afula compared to TA. Concerning parks and homes, in TA the research participants were exposed to low CO concentrations, with no measurable CO concentrations having been recorded in Afula. There was no relationship between the congestion index and CO level.

The calculated percentage time of the subjects’ exposure to CO is presented in Table 3. The exposure to CO in both cities was temporary, extending to less than half the measured time, with most of the events of exposure taking place in city centers adjacent to the main transportation routes. In the other environments, the percentage of the exposure time to CO was either low or zero. A difference in the CO concentrations between the cities was recorded. While the participants were not exposed to CO in Afula’s parks and homes, in TA the participants were exposed to low levels of CO in these environments for 15% to 25% of the measured time.

A difference in the mean LF/HF between the two cities was identified (Table 4). However, the increased mean observed in TA, compared to Afula, was associated with a sizable increased standard deviation.

Higher values of LF/HF, as well as of noise and thermal load, were found in TA. The mean CO levels were higher in Afula due to the high CO concentrations in the city center, albeit with generally-low values (Table 4). By way of contrast, differences in the exposure to noise, and to some extent thermal load, were recorded. The resulting contribution of CO to LF/HF, while significant, appears to be negative compared to the other independent factors. After accounting for noise, thermal load, and traffic congestion, this became marginal (Table 5). The social load also appears to contribute to the prediction of the variability in LF/HF, albeit less so than with noise and thermal load.

This section describes the structure of the functions associating CO concentrations with the frequency domain index of HRV (LF/HF) in the different environments. In the analysis, we accounted for the possible effects of traffic congestion, noise, thermal load, CO, and social load on LF/HF by applying a multiple regression analysis. We separately calculated the slopes of the functions for each environment in each of the two cities (Table 6). In both cities, a significant negative relationship between CO and LF/HF in the residential area was identified. In homes in TA, low levels of CO were measured at times, but the effects of CO on LF/HF in the multiple regressions were not statistically significant. Since no concentrations of CO were recorded in the home and park environments in Afula, no regressions were calculated. Concerning city centers, highly significant results were calculated for Afula, and insignificant results were calculated for TA. Negative slopes between CO and LF/HF characterized the functions for all six environments.

## 4. Discussion

The present study considers CO distribution and its effect on the ANS of young women, as recorded in two Israeli cities of different sizes. A comparison between the two cities—which were of different sizes but similar levels of compactness, and with traffic congestion serving as a main source of pollution—lead to four main conclusions.

1. The levels of CO in these cities are very low.
2. The concentrations of CO are not necessarily affected by the city’s size.
3. Within the range of low concentrations (under 7 ppm per ½ hour), an increased concetration of CO is associated with a decrease in LF/HF, at least in the short term.
4. The ANS balance, as measured using HRV, is mainly affected by environmental factors other than CO, such as noise, transport congestion, thermal load, and social load.

Concerning the first hypothesis, the CO concentrations in most outdoor environments did not exceed 7 ppm per half an hour, and were even close to zero at most times. This, however, excludes small pockets of air pollution with higher levels of CO, in line with a phenomenon identified in a previous study [31]. These CO values are low, even in comparison to other cities around the world. Based on the measurements from stationary stations or satellites, the concentrations of CO in TA were between 0–6 ppm per half an hour, depending on the type of environment. However, previous studies have shown that local measurements conducted in close proximity to the research subjects, performed at several sites in TA, revealed higher concentrations of CO. Some measurements at the main city intersections reached 50 ppm; others, in small pockets like parking lots, even reached as high as 80 ppm [32]. Compared to the measurements performed in selected European cities, these are relatively low concentrations of CO. In European environments, levels of 0.05–0.12 ppm were measured outside of cities. Levels of 17–52 ppm were measured in central city environments, and levels of up to 100 ppm were measured in environmental pockets [33].

These results raise an important question: is it still relevant to monitor global indices of CO concentrations in developed cities polluted mainly by traffic congestion? Instead, it may be more effective to identify specific pockets of CO pollution, monitor them locally, and alert local residents to the CO concentration levels in such sites.

Concerning the effect of city size on the concentrations of CO according to the second hypothesis, three main results stand out. First, people in TA, the larger city, did not accumulate higher levels of CO compared to people in the smaller city of Afula. Second, the CO concentrations in TA are more widely distributed across spaces, including home and park environments, compared to Afula, where CO concentrations were detected almost exclusively in the city center. Third, the exposure time to CO appears to be longer in TA than in Afula. The increased CO concentrations in the more compact city centers, compared to the parks and residential areas in both cities, is possibly related to higher traffic congestion and the area’s morphological structure, compared to the cities’ fringes. This is in accordance with studies conducted in other cities, which placed explanatory weight on factors such as city compactness and size [6]. Our study supports the proposition that increased compactness and congestion in city centers contributes to increased air pollution [6]. Previous studies have pointed to a link between city size and CO concentration. Given that traffic congestion and the morphological characteristics of the tested closed environments have not been accounted for, there is a real possibility that these factors may in fact be the culpable mediators. One can speculate that, apart from city size, the morphology together with local climate conditions will contribute to the resulting CO concentration within the city. The fact is that, unlike CO, the concentrations of other air pollutants such as dust increase in line with traffic congestion [5], and may be attributed to CO’s instability. However, the present study does not account for other pollutants that could support this argument. Further studies of local micro sites would be required in order to isolate the in situ data on variables like traffic congestion and morphological structures, etc. However, we do hypothesize that morphological structures can have a significant effect on CO concentration, more than the intensity of the sources of CO.

As for the effect of CO on LF/HF, as addressed in the third hypothesis, our study shows that an increase in CO concentration, up to 7 ppm, is associated with reduced levels of LF/HF. Only six previous studies employing valid methodology—including CO measurements in the immediate vicinity of the participants—have addressed the association between levels of CO in cities in general and their effect on LF/HF. Four of these studies identified a negative association between the CO concentration and the level of LF/HF [25].

The fact that all six regressions, across the four environments and in two different cities, yielded consistent results lends strong support to the validity of the observed negative relationship between CO and LF/HF when controlling for noise, thermal load, and traffic congestion. However, it should be noted that the other environmental factors accounted for in the present study are associated with a less favorable ANS balance, as previously reported for other health conditions [31]. In addition, our study shows that the other environmental factors are more dominant in explaining the variances in LF/HF. Conflicting results from previous studies concerning the effect of different air pollutants on health have been reported. Refs. [34,35] both proposed that the origins of these discrepancies are related to the measurement methods, which were either personal or ambient. They argued that when personal measurements are performed, NO_2_ emerges as the main contributor to increased health risk. These intervening factors may possibly reverse the negative relationship between low concentrations of CO and LF/HF. In other words, while an increase in CO concentration is associated with decreased LF/HF, other air pollutants and noise may increase the LF/HF ratio, as demonstrated in a recent study by Tang et al. [23]. However, despite the dominant effect of NO_2_, this study aims to highlight the uniqueness of the behavior of CO. The differential correlation coefficients, as related to the different environments, possibly point to the complex relationship between CO and HRV. In residential areas, where the CO is of measurable levels and other environmental variables have a modest effect, the correlation is consistently significant. However, it becomes less significant when these intervening variables become more conspicuous, minimizing the CO effect. The true meaning of the decline in the levels of LF/HF in response to exposure to increased levels of CO (albeit within safe standards) remains a matter of speculation.

It is worth mentioning that despite the expected, and well-documented, positive association between traffic congestion and CO concentration [32], the correlation between CO and LF/HF in our study, albeit marginal, is negative and in line with previous reports [36]. One may speculate that this apparent paradox results from traffic congestion serving as a proxy factor for increased noise, other air pollutants, and the perceived risk of accidents [29,37,38,39,40,41,42].

### Study Limitations

First, this investigation used data collected for two projects employing different HRV sampling procedures. While, in one city, the data were continuously recorded, and then periods of interest related to the specific environments were selected for the current analysis, in the other city the sampling procedure was defined a priori with regard to the targeted environments of interest. Second, although the climate conditions and CO background concentrations were comparable with respect to the two projects, the studies were performed two years apart. Third, a myriad of environmental pollutants possibly affecting both CO concentration and ANS balance were not accounted for in our study. Our ecological approach demonstrates how people are exposed to and are physiologically affected by low levels of CO, which remain low regardless of the city sizes. It appears, however, that a more differentiated approach, focusing on a variety of arenas within the city and capable of isolating in situ traffic congestion and morphological structures, may be more accurate in delineating the role of each variable on human stress. Furthermore, a multivariable approach is mandatory in the study of the effects of pollution and other environmental factors on the ANS balance. Fifth, the study was conducted with young women, who are considered more vulnerable to CO compared to men. There is a need to test more heterogeneous groups of protagonists, including men, older people, and people with health problems. The present study measured the short-term effects of CO on HRV. Only longer time spans may result in more valid outcomes. The index of social load, albeit tested for construct validity, is yet to be verified by other researchers. A few studies have suggested inconsistent results concerning the effect of gender in explaining HRV responses to CO exposure [26].

## 5. Conclusions

The results of this pioneering research suggest, in line with our first and second hypotheses concerning the low concentrations of CO in cities regardless of city size, that the relationship between CO concentration and city size is a complex phenomenon, which is more likely to be more closely related to local morphologies than to city size. We further suggest that the concentrations of CO, regardless of city size, are mostly in the non-noxious range for both cities. These results call for more detailed studies. They show that the global concentrations of CO in cities may be negligible. Instead, local pockets of high concentrations of CO need to be detected and treated in order to avoid any health risk. This leads us to question the current approach to the assessment of CO concentration, and its effect on humans, in developed cities. In line with the third hypothesis concerning the association between CO concentration and levels of LF/HF, it appears that regardless of city size, low levels of CO have a moderating effect on the ANS balance, at least in the short term. This observation has been replicated in other studies using methodologies similar to that employed in the present study. The consequent effect on human general health was not within the scope of the present investigation, and should be evaluated in future studies.

Our study found that the contribution of traffic congestion, noise, and thermal load to the ANS balance is more significant than that of CO per se, when the latter records measurements within the low range. This leads us to conclude that CO monitoring methods for research purposes should employ direct in situ, rather than remote, measurements. Targeting suspected morphological pockets of CO pollution is one example of this approach.

## Figures and Tables

**Table 1:** The study cities’ main characteristics.

City	Population	No. of Cars	Car Congestion ^1^	City Compactness			
				Index	Rank	Cluster	Average Car Age
TA	460,000	287,317	7000	1.47	187	9	4.9
Afula	54,000	17,768	1900	1.68	192	9	6.8

^1^ = Maximum number of cars per hour at the four most crowded entrances to the city. TA = Tel Aviv.

**Table 2:** Mean and standard deviation of the CO concentrations in the selected types of environments in the two cities.

CO_ppm_ in City	TA	Afula
Environments	Mean/S.D.	Mean/S.D.
Center	1.4 (1.3)	4.8 (0.27)
Neighborhood	0.8 (0.7)	0.3 (0.4)
Park	0.2 (0.4)	0.0 (0)
Home	1.2 (1.5)	0.0 (0)

CO = carbon monoxide. TA = Tel Aviv.

**Table 3:** Mean percentage time of CO exposure in the selected environments, by city size.

CO_ppm_	Home		Park		City Center		Residential		Total	
	Afula	TA	Afula	TA	Afula	TA	Afula	TA	Afula	TA
0	100	75	100	86	40	35	82	80	66	57
0.1–2.9	0	25	0	1	11	23	18	14	8	22
3.0–5.9	0	0	0	0	29	13	0	6	14	17
6.0–15	0	0	0	13	20	12	0	0	12	4
Mean	0	0	0	1.8	4.9	3.8	0.3	0.5	1.4	1.5
Maximum									8.1	15.0

CO = carbon monoxide. TA = Tel Aviv.

**Table 4:** Differences in mean (S.D) CO, noise, thermal load and LF/HF, by city size.

Environmental Factor	Mean/S.D. and Difference by City Size		
	Mean TA	Mean Afula	Sig. of Mean Difference
CO	1.3 (2.2)	1.9 (2.4)	0.0001
Noise	81.0 (19)	63.0 (16)	0.0001
Thermal Load	24.2 (5.8)	22.0 (07)	0.0001
Social load	20.8 (14.2)	45 (30)	0.0001
LF/HF	10.9 (10.8)	8.5 (4.9)	0.0001

CO = carbon monoxide. LF/HF = ratio of LF-to-HF power.

**Table 5:** Regression analysis between the environmental factors and the LF/HF in the two cities.

Environmental Factor	B	T	Sig.	Percent Predicted
CO	−0.06	−4.0	0.0001	6
Noise	0.54	31.4	0.0001	42
Thermal load	0.18	11.4	0.0001	18
Social Load	0.10	3.1	0.002	5
Transport congestion	0.37	24.1	0.0001	34

CO = carbon monoxide. LF/HF = ratio of LF-to-HF power.

**Table 6:** The regression slopes between CO and LF/HF, after controlling for traffic congestion, noise, and thermal load, as related to the different environments in the two cities.

City	Environment	B	T	Sig.
Afula	Home	NA	NA	NA
	Park	NA	NA	NA
	Center	−0.09	−2.5	0.04
	Residential	−0.23	−3.1	0.01
TA	Home	−1.15	−1.2	0.24
	Park	−0.26	−1.8	0.05
	Center	−0.01	−0.05	0.9
	Residential	−0.16	−1.9	0.05

NA = not applicable (almost all data for CO are zero). TA = Tel Aviv.

## Data Availability

Not applicable.

## Abbreviations

ANS	Autonomic nervous system
CO	Carbon monoxide
HF	High frequency
HRV	Heart rate variability
LF/HF	Ratio of LF-over -HF power
LF	Low frequency
PPM	parts per million
TA	Tel Aviv
